# Multiple air pollutant exposure and lung cancer in Tehran, Iran

**DOI:** 10.1038/s41598-021-88643-4

**Published:** 2021-04-29

**Authors:** Zahra Khorrami, Mohsen Pourkhosravani, Maysam Rezapour, Koorosh Etemad, Seyed Mahmood Taghavi-Shahri, Nino Künzli, Heresh Amini, Narges Khanjani

**Affiliations:** 1 Neurology Research Center, Kerman University of Medical Sciences, Kerman, Iran; 2 Department of Geography and Urban Planning, Shahid Bahonar University of Kerman, Kerman, Iran; 3 Amol Faculty of Paramedical Sciences, Mazandaran University of Medical Sciences, Sari, Iran; 4 Department of Epidemiology, School of Public Health and Safety, Shahid Beheshti University of Medical Sciences, Tehran, Iran; 5 Department of Epidemiology and Biostatistics, School of Public Health, Isfahan University of Medical Sciences, Isfahan, Iran; 6 Department of Epidemiology and Public Health, Swiss Tropical and Public Health Institute, Basel, Switzerland; 7 University of Basel, Basel, Switzerland; 8 Department of Public Health, Section of Environmental Health, University of Copenhagen, Copenhagen, Denmark; 9 Department of Environmental Health, Harvard T.H. Chan School of Public Health, Boston, MA USA; 10 Environmental Health Engineering Research Center, Kerman University of Medical Sciences, Kerman, Iran; 11 Monash Centre for Occupational & Environmental Health, School of Public Health and Preventive Medicine, Monash University, Melbourne, Australia; 12 Department of Epidemiology and Biostatistics, School of Public Health, Kerman University of Medical Sciences, Kerman, Iran

**Keywords:** Cancer, Environmental sciences, Natural hazards, Diseases, Risk factors

## Abstract

Lung cancer is the most rapidly increasing malignancy worldwide with an estimated 2.1 million cancer cases in the latest, 2018 World Health Organization (WHO) report. The objective of this study was to investigate the association of air pollution and lung cancer, in Tehran, Iran. Residential area information of the latest registered lung cancer cases that were diagnosed between 2014 and 2016 (N = 1,850) were inquired from the population-based cancer registry of Tehran. Long-term average exposure to PM_10_, SO_2_, NO, NO_2_, NO_X_, benzene, toluene, ethylbenzene, *m*-xylene, *p*-xylene, o-xylene (BTEX), and BTEX in 22 districts of Tehran were estimated using land use regression models. Latent profile analysis (LPA) was used to generate multi-pollutant exposure profiles. Negative binomial regression analysis was used to examine the association between air pollutants and lung cancer incidence. The districts with higher concentrations for all pollutants were mostly in downtown and around the railway station. Districts with a higher concentration for NOx (IRR = 1.05, for each 10 unit increase in air pollutant), benzene (IRR = 3.86), toluene (IRR = 1.50), ethylbenzene (IRR = 5.16), p-xylene (IRR = 9.41), o-xylene (IRR = 7.93), m-xylene (IRR = 2.63) and TBTEX (IRR = 1.21) were significantly associated with higher lung cancer incidence. Districts with a higher multiple air-pollution profile were also associated with more lung cancer incidence (IRR = 1.01). Our study shows a positive association between air pollution and lung cancer incidence. This association was stronger for, respectively, p-xylene, o-xylene, ethylbenzene, benzene, m-xylene and toluene.

## Introduction

Lung cancer was the most common cancer worldwide with an estimated 2.09 million cancer cases in the latest, 2018 World Health Organization (WHO) report. It ranked as the first most common cancer for men (31.5 per 100,000) and second most common for women (14.6 per 100,000) after breast cancer. Lung cancer contributed to 18.3% of all cancer deaths worldwide^1^. The age standardized incidence rate (ASR) of lung cancer in 2015, in Iran was 8.52 per 100,000 ranging from 12.37 per 100,000 in West Azerbaijan to 3.84 per 100,000 in Sistan and Bluchestan province; and in Tehran (the country capital) was 8.74 per 100,000, which was more than the incidence in the whole country (Iran)^2^.

Air pollution is likely to be a risk factor for lung cancer. According to WHO, about 24% of the global burden of disease and 23% of global deaths can be attributed to environmental factors^1,3^. Air pollution consists of a complex mixture of particles and gases^4^, in differing amounts, depending on the types of emission sources and atmospheric conditions. Typically, air pollution consists of a range of pollutants such as soot, particulate matter (PM) ≤ 10 µm (PM_10_), and PM ≤ 2.5 µm (PM_2.5_), nitrogen dioxide (NO_2_), sulfur dioxide (SO_2_), carbon monoxide (CO), ozone (O_3_) and volatile organic compounds (VOCs)^5^. The latest International Agency for Research on Cancer (IARC) report has mentioned PM_2.5_ as a risk factor for lung cancer^6^ and the ELAPSE study in Europe showed that long-term ambient PM_2.5_ exposure may cause lung cancer even at concentrations lower than current European threshold values and WHO Air Quality Guidelines^7^.

Humans are simultaneously exposed to a complex mixture of air pollutants; therefore, many researchers have investigated a multiple-pollutant approach for assessing air pollution exposure^8^, because in single pollutant models, it is not clear if an observed association reflects the effect of the specific pollutant under study, or the effect of coinciding pollutants^9^. However, there is no consensus on the method used for measuring multiple ambient air pollutants simultaneously. Previous review studies have evaluated multiple pollutants by using methods that can be classified into three groups; dimension reduction, variable selection, and grouping of observations^4,8,10,11^. However, Caban-Martinez, et al.^12^ and Kolpacoff, et al.^13^ have used latent profile analysis (LPA) to identify subgroups of cancerous patients with different multi-pollution profiles. LPA is a probabilistic or model-based technique and is a variant of the traditional cluster analysis, which better handles outliers and unequal cluster sizes^14^. This method enables identifying possible unobservable subgroups, or latent classes in a population using a number of related observable variables. Using this method, complex relations between groups of risk factors and disease outcomes, such as cancer, which may not be best explained by a single pollutant model, can be better understood. It also reduces the dimensionality of exposure data and decreases the burden of multiple testing, while enhancing the power of statistical analysis^13^.

Knowledge about geographical patterns of multiple pollution helps policy makers to target high risk regions for more intense interventions. Air pollution exacerbates the health disparity among socioeconomic groups, because usually the poor socio-economic areas are more polluted^15,16^. The relation between air pollution exposure and health outcomes can also be theoretically modified by socioeconomic status, through causing differences in access to medical care and healthy diet, and also by biological factors, such as age and psychological stress. However, the hypothesis that residents with a low socioeconomic position face more severe consequences from air pollution is debated^16^.

In the current study, we aimed to examine the association between single and multiple ambient air pollutants and lung cancer incidence in Tehran, Iran.

## Methodology

### Research location

This study was conducted in Tehran, a megacity which is the most populous city in Iran, with a residing population of about 9 million and a daytime population of over 14 million people. According to the World Population Review report, Tehran's 2020 population is now estimated to be 9,134,708. Tehran is the most populous city in Iran and Western Asia, and has the third-largest metropolitan area in the Middle East. It is ranked 24th in the world by the population of its metropolitan area. Tehran’s area extend is about 730 km^2^ and consists of 22 municipal districts with different concentrations of ambient air pollutants^17^.

### Data sources

#### Lung cancer data

Totally, 1850 patients residing in Tehran were diagnosed with lung cancer (Trachea, Bronchus and Lung) between 2014 and 2016. The latest address of these patients' residence in Tehran was inquired from the Cancer Department of the Ministry of Health of Iran. The officials of the cancer registry claimed that the recorded addresses are more than 90% accurate.

The geographical coordinates (longitude and latitude) of the participants' residential addresses were determined according to the address of the patients' residence and was marked on the GIS map of Tehran.

#### Exposure assessment

The annual mean concentrations of PM_10_, SO_2_, NO, NO_2_, NO_X_, benzene, toluene, ethylbenzene, *m*-xylene, *p*-xylene, *o*-xylene (BTEX), and total BTEX in the 22 districts of Tehran were inquired from previously developed land use regression (LUR) models. The LUR models for PM_10_, SO_2_, NO, NO_2_ and NO_X_ in Tehran were developed based on measurements conducted at 23 sites in Tehran in 2010^18,19^. The models for volatile organic compounds (VOCs) were developed based on measurements at 179 sampling sites from April 2015 to May 2016^20,21^.

#### Confounding covariates

Population-based data was extracted from the Urban Health Equity Assessment and Response Tool (Urban HEART-2), which has been conducted in 22 districts of Tehran and is a data repository that collected many district-level variables, such as population density, per capita urban green space, smoking rates and life expectancy in 2011. A detailed description of the Urban HEART-2 study can be found elsewhere^22^**.**

The socio-economic development situation of the 22 districts of Tehran was extracted from a study conducted by Sadeghi et al.^23^. In brief, sixteen economic and social indicators were incorporated to estimate the level of development in the 22 districts of Tehran, based on Exploratory Factor Analysis (EFA), Principal Component Analysis (PCA). This multivariate statistical technique is used to reduce the number of variables in a dataset into a smaller number of “dimensions” that explains most variations in the dataset using a few estimated substitutional latent variables^23^.

In Sadeghi et al.’s study, the variables used for estimating the social dimension included adult literacy level (among the 30–59 year olds), elderly literacy level (60 years and older), the proportion of university graduates in the total population, the proportion of the males with university education, the proportion of females with university education, and the percent of population that uses the internet. The variables used for estimating the economic part, included women's economic participation rate, the proportion of employees with high-rank jobs, the proportion of households with cars, the proportion of households with computers, indicators of household access to public facilities, the ratio of households who own their home, the proportion of homes with civil standards, the average price per square meter of residential building land, the average selling price per square meter of residential building area and the average monthly rent per square meter of residential area for each district. A detailed description of these variables can be found elsewhere^23^. A higher socio-economic development score showed a higher socio-economic level. This variable did not have a specific unit, its minimum was 36.6 and maximum was 67.4^23^.

### Statistical analyses

Latent profile analysis (LPA) was used to make multiple-pollution profiles^14^. A series of LPAs was performed, ranging from two to seven latent profiles. The 12 air pollutants (PM_10_, SO_2_, NO, NO_2_, NO_X_, benzene, toluene, ethylbenzene, *m*-xylene, *p*-xylene, *o*-xylene and TBTEX) were used as LPA indicators. The grand mean centering of all 12 pollutants were used in the analysis to facilitate interpretation. Grand-mean centering subtracts the overall mean from a variable^24^. To identify the best fitting model, measures of relative statistical fit and the interpretability of their profile structure were used. Models with low values in AIC, BIC, aBIC metrics, and a significant Bootstrap Likelihood Ratio Test (BLRT) were preferred^25^. The absolute value of log likelihood is not recommended to be used for model selection, because this value gradually improves by adding more parameters to the model. Entropy was also evaluated for each model; and values closer to 1 suggest a higher discrimination of the latent classes^26^. In addition, the interpretability and parsimony of the candidate models were compared. Models with profiles including less than 5% of the class size are considered spurious^27^, and are not acceptable. Data preparation was done in Stata version 14. LPA was performed using Mplus version 7.4 (Muthen & Muthen, 1998–2015) mixture modeling procedure, with the robust maximum likelihood (MLR) estimator. Missing data were addressed using full information maximum likelihood (FIML). To examine how the various multiple-pollution profiles differ in terms of each air pollution component, one-way ANOVA and post hoc follow-up tests were used.

Kolmogorov Smirnov tests were used to test the normality of the pollutants and because the data was normally distributed, the Pearson’s correlation test was used to estimate the correlation between pollutants. As the number of lung cancer cases was over-dispersed, negative binomial (NB) regression was performed to estimate the incidence rate ratios (IRR) and their 95% confidence intervals (CI) for each air pollutant and multiple pollution profiles, adjusted for age, sex, smoking at district level.

In order to calculate the population attributable fraction (PAF), the risk estimates for air pollutants were obtained from the results of negative binomial (NB) regression analysis.

The PAF for air pollutants as continuous variables, were estimated using the following equation^28^:

$$PAF=\frac{\mathrm{exp}\left[Ln\left(RRunit\right) \times \overline{x }\right]-1}{\mathrm{exp}[Ln\left(RRunit\right) \times \overline{x }]}$$

In this equation, RR_unit_ is the relative risk for each one unit increment in exposure to the air pollutant and $\overline{\chi }$ is the average of exposure.

Statistical analyses were performed using Mplus version 7.4, Stata version 14 (Stata Corp LLC; College Station, TX, USA) and ArcGIS version 10.8.

As the data was inquired in aggregated form and anonymously, informed consent from individuals or their family was not required. This data is not publicly available, but can be inquired by formal request in aggregated and/or anonymous form from the Ministry of Health of Iran. The Ethic approval code of this project was IR.KMU.REC.1398.230. All methods were carried out in accordance with relevant guidelines and regulations.

## Results

**Figure 1:**
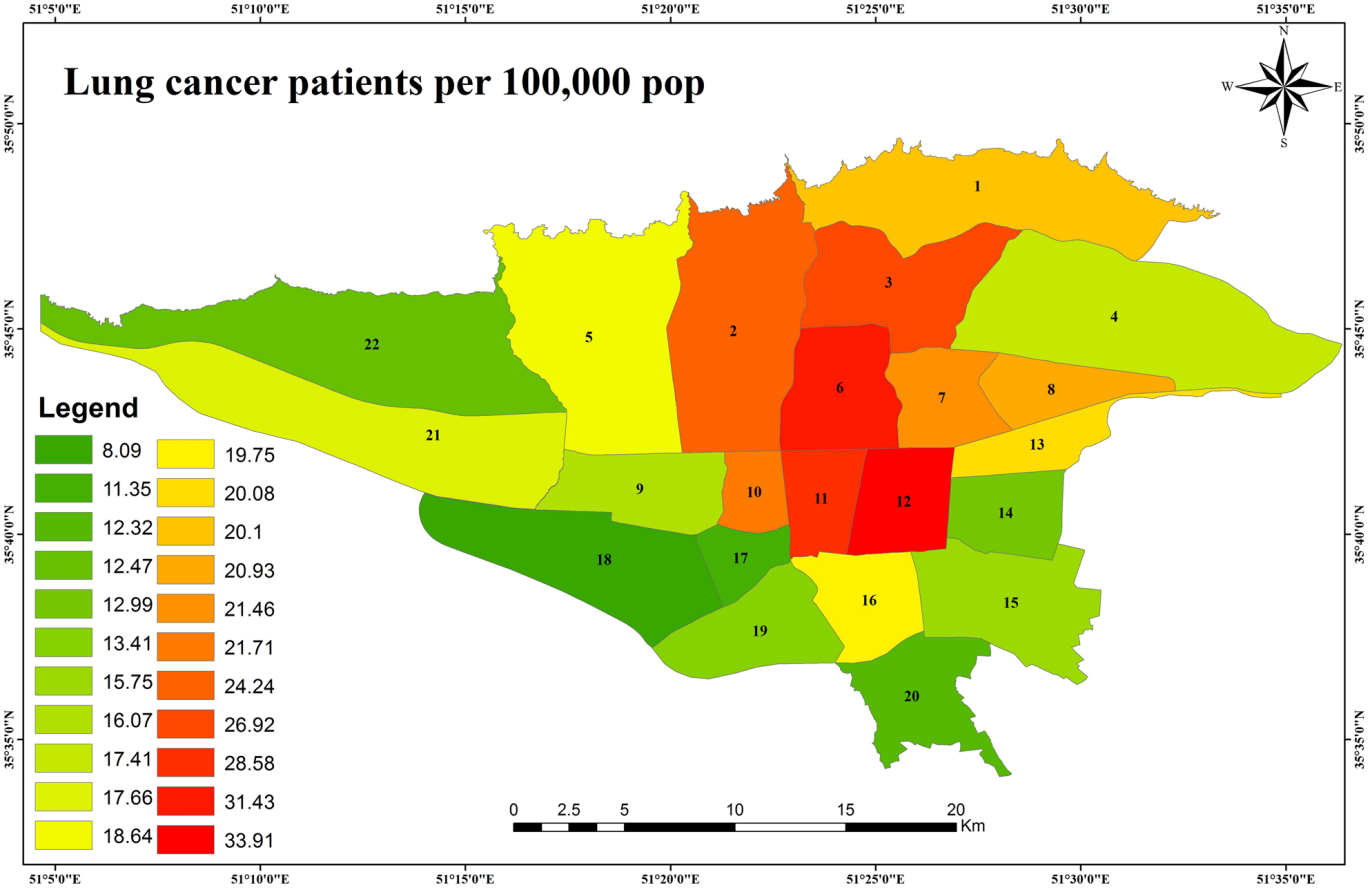
Spatial distribution of lung cancer patients (number of cases in 100,000) in different areas of Tehran in 2014–2016.

Basic information about the area under study is shown in Table 1. The total number of lung cancer cases in 2014–2016 was 1850 in all districts of Tehran. We had to exclude the data of subjects who lived in remote suburbs of Tehran, which air pollutants estimation was not possible. Eventually, 1653 cases entered the final analyses. The distribution of lung cancer patients in different districts of Tehran is shown in Fig. 1. The highest number of patients per 100,000 populations was in regions 12, 6 and 11, respectively.

**Table 1:** Description of the study area, air pollution level, and district-level covariates.

Number of districts	22
Number of lung cancer patients	1653
**Age of patients (year)**	
Median (1st–3st quartile range)	65.5 (60–71)
**Gender of patients (male)**	
Percent (number)	70.1 (1158)
**Annual mean air pollutants**	Median (1st–3st quartile range)
PM_10_ (μg/m^3^)	85.42 (65.5–99.9)
SO_2_ (ppb)	50.82 (32.9–61.7)
NO_2_ (ppb)	45.46 (41.6–50.2)
NO (ppb)	74.02 (55.6–98.5)
NO_X_ (ppb)	112.21 (92.1–137.2)
Benzene (μg/m^3^)	9.05 (8.2–9.9)
Toluene (μg/m^3^)	26.35 (23.6–29.9)
Ethylbenzene (μg/m^3^)	6.49 (5.8–7.2)
*p*-xylene (μg/m^3^)	6.04 (5.6–6.6)
*o*-xylene (μg/m^3^)	6.60 (5.9–7.6)
*m*-xylene (μg/m^3^)	11.85 (10.4–13.4)
TBTEX (μg/m^3^)	64.68 (58.65–70.90)
**District level variables**	Median (1st–3st quartile range)
Total population (persons)	319,443 (263,536–421,225)
Life expectancy (year)	75.9 (75.4–76.7)
Population density (per km^2^)	158.5 (127.5–208.1)
Urban green space, per capita (m^2^ per 1000 people)	13.9 (10.7–18.0)
Socioeconomic status*	49.95 (44.21–53.34)
Smoking prevalence in men (%)	15.7 (15.1–16.7)
Smoking prevalence in women (%)	0.79 (0.67–1.18)

*Socio-economic status score according to the 16 variables mentioned in the method section. This variable does not have a unit. The lowest value of this score was 36.6 and the highest was 67.4.

**Figure 2:**
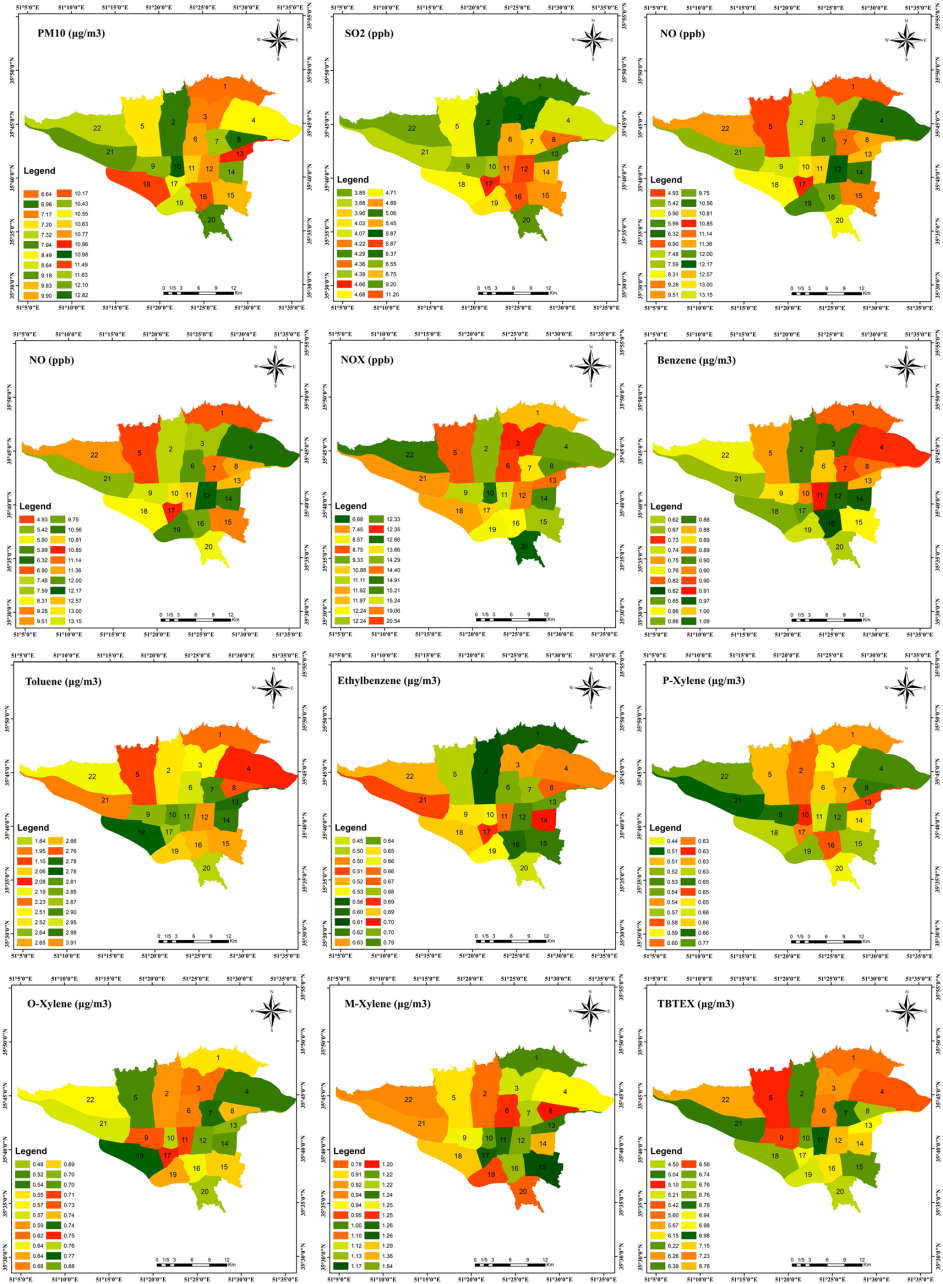
Spatial distribution and average levels of ambient air pollutants in different areas of Tehran in 2014–2016.

The spatial distributions and average levels of ambient air pollutants in different districts of Tehran are shown in Fig. 2. Districts with higher concentrations for pollutants were mostly in downtown (district 6, 7, 11, 12 and 14(, and around the railway (district 16 and 17), and a few of the southern districts of the city (district 18). District 16 had the highest concentration of SO_2_ and district 9, 2 and 6 had the highest concentration of NO, NO_2_ and NOx pollutants during these years. District 12 had the highest concentration of VOC pollutants.

**Figure 3:**
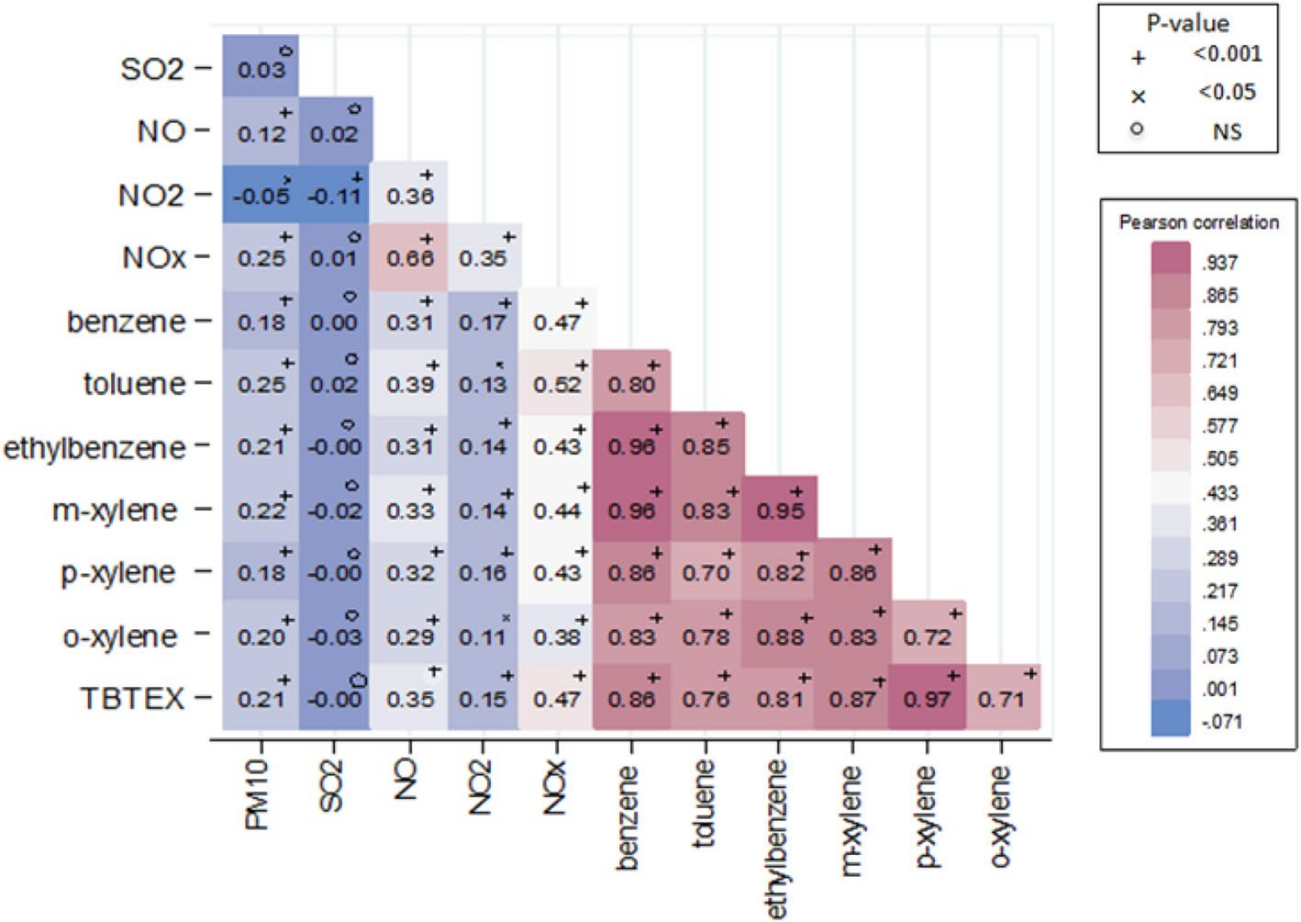
Pearson correlation matrix of air pollutants in the 22 districts of Tehran in 2014–2016.

As indicated in Fig. 3, there was a strong correlation (Pearson's r) between pollutants, most notably for benzene compounds (benzene, toluene, ethylbenzene, m-xylene, p-xylene, o-xylene and TBTEX) (p-value < 0.001). The positive correlation between NO and NOx was weaker (p-value < 0.001).

**Figure 4:**
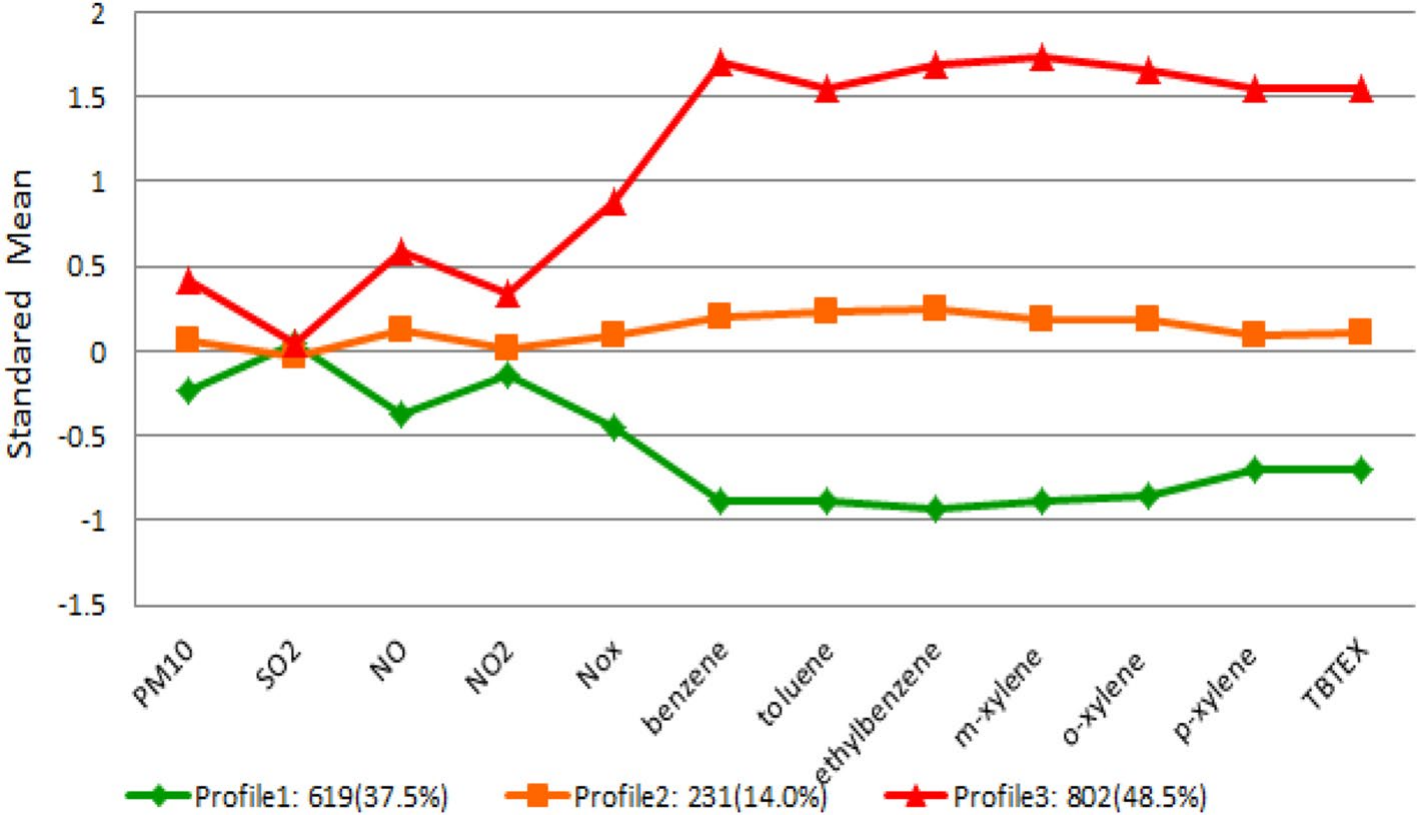
Standard mean values of pollutants in the three latent profiles in different areas of Tehran in 2014–2016.

Fit indices for the different LPA models are displayed in Table 2. All solutions provided acceptable classification accuracy, as indicated by entropy values close to 1. Although models with four, five, six, and seven latent class profiles had lower AIC, BIC, and aBIC than two and three latent class profiles, these models included classes with less than 1% of the sample. Therefore, the three latent profile model was preferred. The multi-pollution profiles are shown in Fig. 4. Profile 1 had the lowest scores for all pollutants, except SO_2_. We labeled this profile as “low multiple-pollution”. Profile 3 had the highest scores of all pollutants. We labeled this profile as “high multiple-pollution”. Profile 2 was in between and was labeled “medium multiple-pollution”.

**Table 2:** Fit indices for different models with number of profiles ranging from 2 to 7.

	Log-likelihood	AIC	BIC	aBIC	Entropy	BLRT
2 Profile	-70,802.1	141,678.2	141,878.3	141,760.8	0.93	6922.7***
**3 Profile**	**-69,084.9**	**138,269.8**	**138,540.3**	**138,381.4**	**0.92**	3434.3***
4 Profile	-67,326.8	134,779.7	135,120.5	134,920.4	0.94	3516.1***
5 Profile	-66,087.4	132,326.8	132,738.0	132,496.5	0.96	2478.8***
6 Profile	-65,394.4	130,966.8	131,448.3	131,165.5	0.94	1386.1***
7 Profile	-64,790.5	129,785.1	130,336.9	130,012.8	0.95	1469.5***

*Note* AIC Akaike’s Information Criterion, BIC Bayesian Information Criterion, aBIC Sample Size Adjusted BIC, and BLRT bootstrap likelihood ratio test. ***P value < 0.001. Models with a low AIC, BIC, and aBIC value and high Entropy value as well as significant Bootstrap Likelihood Ratio Test (BLRT) were preferred.

Bolded Fit Indices indicate that the 3 profile is optimal latent profile model, The selected (3 profile) has been bolded.

Summary statistics for each pollutant in different profiles are shown in Table 3. There was a significant difference between the means of all pollutants in the three profiles, except SO_2_.

**Table 3:** The mean of air pollutants in different profiles.

Pollutant	Profile	Mean	SD	F	P-value
PM_10_	Profile 1	69.76	41.74	39.30	< 0.001
	Profile 2	85.08	52.11		
	Profile 3	101.86	57.57		
SO_2_	Profile 1	52.42	37.78	1.22	0.295
	Profile 2	49.87	32.54		
	Profile 3	52.89	36.56		
NO	Profile 1	64.95	43.23	104.96	< 0.001
	Profile 2	93.39	53.37		
	Profile 3	118.36	64.64		
NO_2_	Profile 1	46.65	16.42	19.22	< 0.001
	Profile 2	49.46	16.32		
	Profile 3	54.78	21.23		
NO_x_	Profile 1	97.19	40.54	197.56	< 0.001
	Profile 2	138.21	65.09		
	Profile 3	195.08	107.85		
Benzene	Profile 1	7.09	1.15	2132.15	< 0.001
	Profile 2	9.79	1.10		
	Profile 3	13.57	2.15		
Toluene	Profile 1	20.76	3.63	1589.85	< 0.001
	Profile 2	29.16	4.42		
	Profile 3	38.94	5.72		
Ethylbenzene	Profile 1	5.01	0.85	2664.12	< 0.001
	Profile 2	7.07	0.76		
	Profile 3	9.60	1.06		
M-xylene	Profile 1	8.93	1.57	2371.73	< 0.001
	Profile 2	13.01	1.63		
	Profile 3	18.87	3.21		
O-xylene	Profile 1	5.20	0.91	1694.88	< 0.001
	Profile 2	7.42	1.28		
	Profile 3	10.59	1.69		
P-xylene	Profile 1	5.00	0.68	892.24	< 0.001
	Profile 2	6.48	0.68		
	Profile 3	9.21	3.04		
TBTEX	Profile 1	51.76	7.89	938.71	< 0.001
	Profile 2	69.73	8.38		
	Profile 3	102.18	35.34		

Table 4 shows the IRR estimates by single-pollutant and multiple-pollutant multivariable negative binomial regression models, adjusted for age, gender, socioeconomic status, life expectancy and smoking prevalence. In single-pollutant models, p-xylene, o-xylene, ethylbenzene, benzene, m-xylene and TBTEX were significantly associated with increased lung cancer incidence in model 3, which was adjusted for age, gender, socioeconomic status, life expectancy and smoking prevalence.

**Table 4:** The estimated incidence rate ratios using negative binomial regression analyses for the effect of each 10 unit increase in air pollutants on lung cancer incidence in the districts of Tehran.

Pollutant	Model 1		Model 2		Model 3	
	RR (95% CI)	P-value	RR (95% CI)	P-value	RR (95% CI)	P-value
Single-pollutant						
PM_10_ (μg/m^3^)	0.97 (0.89–1.05)	0.484	0.92 (0.84–1.002)	0.056	0.99 (0.86–1.14)	0.919
SO_2_ (ppb)	0.93 (0.86–1.006)	0.074	0.93 (0.86–1.01)	0.124	0.98 (0.91–1.05)	0.591
NO_2_ (ppb)	1.19 (1.04–1.37)	0.011	1.17 (1.008–1.36)	0.039	1.04 (0.91–1.19)	0.502
NO (ppb)	1.01 (0.96–1.06)	0.583	0.98 (0.92–1.05)	0.680	1.04 (0.99–1.09)	0.066
NO_X_ (ppb)	1.04 (1.008–1.08)	0.016	1.04 (0.99–1.09)	0.064	1.05 (1.02–1.08)	0.001
Benzene (μg/m^3^)	2.15 (0.65–7.08)	0.206	1.50 (0.37–6.05)	0.567	3.86 (1.55–9.60)	0.004
Toluene (μg/m^3^)	1.14 (0.87–1.50)	0.326	1.03 (0.75–1.42)	0.813	1.50 (1.19–1.88)	< 0.001
Ethylbenzene (μg/m^3^)	1.43 (0.29–6.88)	0.653	0.58 (0.08–3.93)	0.583	5.16 (1.23–21.57)	0.025
P-xylene (μg/m^3^)	2.79 (0.46–16.84)	0.261	1.29 (0.12–13.53)	0.827	9.41 (2.06–42.97)	0.004
O-xylene (μg/m^3^)	1.18 (0.29–4.83)	0.809	0.54 (0.10–2.74)	0.463	7.93 (1.74–36.21)	0.007
M-xylene (μg/m^3^)	1.23 (0.58–2.59)	0.580	0.83 (0.33–2.04)	0.690	2.63 (1.31–5.33)	0.007
TBTEX (μg/m^3^)	1.08 (0.94–1.24)	0.231	1.03 (0.87–1.22)	0.704	1.21 (1.08–1.34)	0.001
Multi-pollutant						
Profile 1 (low pollution)	Ref		Ref		Ref	
Profile 2 (moderate pollution)	1.006 (0.99–1.01)	0.165	1.009 (1.001–1.01)	0.019	1.001 (0.99–1.09)	0.897
Profile 3 (high pollution)	1.01 (1.001–1.01)	0.018	1.01 (1.009–1.02)	< 0.001	1.01 (1.004–1.02)	0.002

Model 1: Adjusted for age and gender.

Model 2: Adjusted for age, gender and urban green space, per capita (m^2^ per 1000 people).

Model 3: Adjusted for age, gender, socioeconomic status, life expectancy and smoking prevalence.

The incidence rate ratio of lung cancer is estimated for each 10 unit increase in pollutants.

In multi-pollutant models, the high multiple-air-pollutants profile was associated with higher lung cancer incidence when compared with the low multiple-air-pollutants profile.

**Figure 5:**
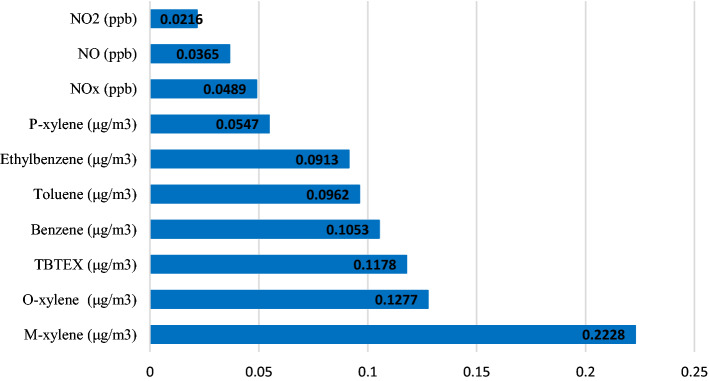
Estimated fraction of all lung cancer incidence attributable to each 1 unit increase in air pollutants in Tehran in 2014–2016.

The fraction of cancers attributable to air pollutant can be seen in Fig. 5. The highest fractions belong to m-xylene, o-xylene, and TBTEX.

## Discussion

This study was the first to investigate the effect of single and multiple ambient air pollutants on lung cancer in Iran. The findings suggest that ambient air pollutants, especially p-xylene, o-xylene, ethylbenzene, benzene, m-xylene and TBTEX were associated with lung cancer. Previously several studies have also shown a strong association between air pollution and respiratory mortality^29,30^ and respiratory diseases^31,32^, including chronic obstructive pulmonary disease (COPD), asthma, bronchitis, and decreased lung function^33^. Recently, some studies have shown the association between air pollution and lung cancer as well^3^.

Iran’s national cancer registry data indicates an approximately sevenfold increase in the trend of lung cancer incidence over a 27-year span (1990 to 2016), in the whole country and in the capital city, Tehran^34^. In the past, lung cancer had been mainly attributed to direct tobacco smoke exposure. However, its increased incidence in never-smokers in the recent years shows that there are other risk factors that need to be discovered^35^. Some of the probable risk factors for lung cancer in never smokers could be environmental pollutants, such as air pollution, occupational carcinogens, radon and infections^36^. In Taiwan, air pollution was related with the incidence of lung cancer in never-smokers^37^; and the result of lung cancer screening programs in China and the United States in 2018 showed that the incidence of lung cancer in never smokers was significantly higher in China than the United States. Their data suggested that inclusion of ambient air pollution could improve the lung cancer risk models, especially for non-smokers^38^.

Air pollutants have been reported to be correlated in many studies. Faridi et al. reported positive correlations between PM_2.5_, PM_10_, NO_2_, SO_2_, CO and O_3_ in Tehran^39^, and another study from Los Angeles County, also reported correlations between multiple ambient air pollutants^40,41^. Studies from Spain have shown positive correlations between PM_10_ and PM_2.5_ and between nitrogen oxides (NO_2_ and NO). The correlations between nitrogen oxides (NO and NO_2_) and particulate matter (PM_10_) is probably due to the common sources of these pollutants that are traffic, heating systems, industries, and other combustion processes^42,43^. High spatial correlations between exposure variables preclude the possibility to do multivariate adjusted analysis in air pollution studies. In the present study, because of the high correlation between pollutants, we used LPA models to investigate the association between multiple -pollutants and lung cancer incidence. Exposure profile modelling for multiple exposures has been used in previous epidemiologic studies on health outcomes such as blood pressure^44^, low birth weight^40^, total mortality^45^, respiratory mortality^46^, and lung cancer in nonsmokers^47^.

This method enables identifying possible unobservable subgroups, or latent classes, in a population using a number of related exposure variables and can help better understand the complex relations between risk factors and health outcomes, such as cancer, that may not be best explained by a single exposure^13^.

The concentration of BTEX in Tehran ambient air between 2005 and 2018 was estimated in a meta-analysis and risk assessment conducted by Abtahi et al. The rank of BTEX concentrations was benzene (149.18 µg/m^3^: 31%) > o-xylene (127.16 µg/m^3^: 27%) > ethylbenzene (110.15 µg/m^3^: 23%) > toluene (87.97 µg/m^3^: 19%). While in the present study toluene, *m*-xylene and benzene had the highest concentrations among VOCs^20^.

The primary sources of benzene and toluene in the ambient air of Tehran includes both mobile and stationary sources of emission. According to the results of Abtahi et al., the pooled concentrations of benzene (149.18 µg/m^3^) and o-xylene (125.57 µg/m^3^) in Tehran were higher than those in other regions around the world such as Ontario (Canada), Orleans (France), Bari (Italy), Kuala Lumpur (Malaysia) and Beijing; and the population residing in Tehran is at a considerable risk of exposure to carcinogens^48^. The inversion phenomenon, fossil fuel consumption of old vehicles, low-quality fuel, population congestion, and high-traffic highways, and the existence of several factories in the south of Tehran such as iron and steel industries, are other reasons for the high level of BTEX in Tehran city^48,49^.

In the present study, about 70% of lung cancer patients were men and the prevalence of smoking among men was about 16%. But even after adjusting for smoking prevalence, the effect of air pollutants on lung cancer was significant.

Su et al. conducted an ecological study about ambient air pollution and all cancer incidences in Taiwan. Their results showed positive correlations between PM_2.5_ SO_2_, NO_x_, and O_3_ levels and age-adjusted total cancer incidence rates^50^; and a study conducted on data from 2002 and 2011 in Brazil showed that traffic density and NO_2_ were associated with an increased incidence of respiratory cancers^51^. In a large population of 16,209 Norwegian men, after a 27-year follow-up the risk ratios for developing lung cancer attributed to NO_x_ and SO_2_ exposure were 1.08 (CI 95% = 1.06–1.11) and 1.03 (CI 95% = 0.77–1.38), respectively^52^. A cohort study conducted from January 1998 to December 2009 in four Northern Chinese cities including Tianjin, Shenyang, Taiyuan, and Rizhao, showed that the combined effect of NO_2_ and PM_10_ resulted in a significant increase in mortality from lung cancer^53^. Bai et al. in a population-based cohort study in Ontario, Canada (2001–2015) showed positive associations between lung cancer incidence with PM_2.5_ (hazard ratio [HR] = 1.02 [95% CI: 1.01–1.05] per 5.3 μg/m^3^) and NO_2_ (HR = 1.05 [95% CI: 1.03–1.07] per 14 ppb), and each ~ 5 μg/m^3^ increase in outdoor PM_2.5_ concentration was associated with a 2% (95% CI: 1%–5%) increased risk of lung cancer. However, no associations were observed for O_3_ or O_x_ and lung cancer^54^. In our analysis, NO_2_ was associated with an increased risk of lung cancer as well. Also a meta-analysis of 14 studies showed that the pooled risk of PM_2.5_ and PM_10_ for lung cancer mortality was respectively RR: 1.14, CI95%:1.07–1.21 and RR: 1.07, CI95%:1.03–1.11^55^.

A population-based cohort study from the Korean National Health Insurance Service (NHIS) database on 2006–2007 data showed that they did not find an increased risk of lung cancer with higher exposure to PM_10_ or NO_2_, in average concentrations of PM_10_ = 60.9 µg/m^3^ and NO_2_ = 32.1 ppm^56^. The largest population-based case–control study of lung cancer among never-smoking females in Xuanwei and Fuyuan, China, done by Song et al. showed that a cluster of 25 PAHs had the strongest association with lung cancer [OR = 2.21; 95% CI = 1.67–2.87); and nitrogen dioxide (NO_2_) was also directly related with lung cancer (OR = 2.06; 95%CI = 1.19,3.49). However, neither benzo (a) pyrene (BaP) nor PM_2.5_ were associated with lung cancer in the multipollutant models^57^. Studies in Canada^58,59^, nine European countries^60^, the UK^61^ and the US^62^ also found that PM_2.5_, nitrogen oxides, nitrogen dioxide, and sulfur dioxide were associated with greater risk of lung cancer^63^. The ESCAPE study based in nine European countries concluded for every 10 µg/m^3^ increase in PM_10_, the risk of lung cancer increases by 22%^60^.

### Strengths and limitation

An important limitation of our study was the short time interval between the recorded pollutants and lung cancer incidence data. However, this was the latest available data about lung cancer in Tehran, at the time we started our study. Also, the study design was ecologic with no individual-level data, and the PAF would have been better estimated using individual-level data with adjustments for important confounding covariates. However, the results of this study still have important implications for public health, underscoring the need to reduce air pollution.

Tehran includes 22 districts, and the number of women with lung cancer was small in some districts, which prevented us to perform further analyses in gender subgroups. However, gender prevalence was adjusted in the analyses. Also, pathological data about cancer cell types was not available, and this prevented us to perform a separate analysis based on pathological type.

A novelty of our study was estimating the simultaneous effect of several air pollutants on lung cancer incidence. This helps to have a holistic picture of the effect of complex air pollution mixtures on human disease.

## Conclusion

This is the first study to examine the associations between multiple air pollutants on lung cancer incidence in Iran. The findings suggest that lung cancer was associated with ambient air pollution in Tehran, and this association was stronger for p-xylene, o-xylene, ethylbenzene, benzene, m-xylene and TBTEX. Air pollution is a serious problem in Tehran, and decreasing the concentrations of air pollutants should be a key goal for policy makers to reduce the number of lung cancer cases in Tehran.
